# Histological and functional characterization of 3D human skin models mimicking the inflammatory skin diseases psoriasis and atopic dermatitis

**DOI:** 10.1242/dmm.050541

**Published:** 2024-01-22

**Authors:** Jasmin Scheurer, Birgit Sauer, Jule Focken, Martina Giampetraglia, Annika Jäger, Christian M. Schürch, Bettina Weigelin, Birgit Schittek

**Affiliations:** ^1^ Department of Dermatology, University Hospital Tübingen, 72076 Tübingen, Germany; ^2^ Department of Pathology and Neuropathology, University Hospital and Comprehensive Cancer Center Tübingen, 72076 Tübingen, Germany; ^3^ Department of Preclinical Imaging and Radiopharmacy, Eberhard Karls University, 72076 Tübingen, Germany; ^4^ Cluster of Excellence iFIT (EXC 2180) “Image-Guided and Functionally Instructed Tumor Therapies”, Eberhard Karls University Tübingen, 72076 Tübingen, Germany

**Keywords:** 3D human skin model, *Staphylococcus aureus*, Atopic dermatitis, Psoriasis

## Abstract

Three-dimensional (3D) human skin equivalents have emerged as valuable tools in skin research, replacing animal experimentation and precluding the need for patient biopsies. In this study, we advanced 3D skin equivalents to model the inflammatory skin diseases atopic dermatitis and psoriasis by cytokine stimulation, and were successful in integrating TH1 T cells into skin models to develop an immunocompetent 3D psoriasis model. We performed in-depth histological and functional characterization of 3D skin equivalents and validated them in terms of tissue architecture, pathological changes, expression of antimicrobial peptides and *Staphylococcus aureus* colonization using 3D reconstruction by multiphoton microscopy and phenotyping by highly multiplexed ‘co-detection by indexing’ (CODEX) microscopy. We show that our skin equivalents have a structural architecture with a well-developed dermis and epidermis, thus resembling human skin. In addition, the skin models of atopic dermatitis and psoriasis show several phenotypic features of inflammatory skin disease, including disturbed epidermal differentiation and alterations in the expression of epidermal barrier genes and antimicrobial peptides, and can be reliably used to test novel treatment strategies. Therefore, these 3D equivalents will be a valuable tool in experimental dermatological research.

## Teaser

**Summary:** Distinct 3D human skin models with phenotypic features of the inflammatory skin diseases psoriasis and atopic dermatitis can be reliably used to test novel treatment strategies.

## INTRODUCTION

Mouse models are widely used and essential for the study of skin physiology, skin diseases and the development of new treatment strategies. However, a major problem is that they inadequately represent human skin, as the skin structure and immune cell composition differ significantly from humans (Mestas and Hughes, 2004; Pasparakis et al., 2014; Salgado et al., 2017). Comparative transcriptome analysis revealed even that only 30% identity among human and mouse skin-associated genes exists (Gerber et al., 2014). Here, three-dimensional (3D) human skin equivalents (HSEs) are attractive tools that better represent the morphology and physiology of human skin. Over the past few decades, a variety of protocols have been developed to generate HSEs. In general, complete HSE models consist of a dermal compartment and the epidermis with stratified, differentiated keratinocytes and a well-developed stratum corneum. They can be generated using genetically modified cells, patient-derived cells or induced pluripotent stem cells (Stanton et al., 2022). In addition, HSEs are highly customizable and can vary significantly in complexity depending on the desired application of the model (Stanton et al., 2022). It is possible to integrate different types of immune cells such as Langerhans cells, macrophages and T cells into HSEs (Facy et al., 2005; Lorthois et al., 2017; van den Bogaard et al., 2014), which allows for the study of immune cells in human skin models. Even adnexal skin structures such as hair follicles or sweat glands have been integrated in HSEs (Stanton et al., 2022). Furthermore, microfluidic culture devices using bioreactor platforms called ‘skin-on-a-chip’ were developed to simulate the vascular structures and blood flow of human skin (Ali et al., 2015; Niehues et al., 2018; van den Broek et al., 2017).

In recent years, particularly great progress has been made in the development of 3D human skin models that mimic inflammatory skin diseases such as psoriasis or atopic dermatitis (AD). Psoriasis and AD are common inflammatory skin diseases that affect 3-10% of individuals worldwide, with an increasing trend every year (Chen et al., 2022). Psoriasis and AD are both triggered by a pathological T cell immune response, but the response is opposite in both diseases. Psoriasis is largely driven by T helper (TH) 1 (TH1) and TH17 cells with the involvement of IL-17A, TNF-α and IL-22, whereas AD is associated with infiltration of TH2 cells in particular (Guttman-Yassky and Krueger, 2017; Nestle et al., 2009). In both diseases, epidermal keratinocytes respond to T cell-derived cytokines with impaired epidermal differentiation. These responses include the formation of a thickened prickle cell layer (acanthosis), a thickened horny layer (hyperkeratosis) with nucleated keratinocytes due to disturbed keratinization (parakeratosis), and edema between keratinocytes (spongiosis) (Schabitz et al., 2021). In psoriasis, moreover, the cytokines IL-17, IL-22, TNF-α and IFN-γ promote the secretion of antimicrobial peptides (AMPs) such as human β-defensins and the cathelicidin LL-37 (encoded by *CAMP*) by keratinocytes (Takahashi and Yamasaki, 2020). AMPs are not only natural antibiotics that directly kill microorganisms: they are also produced by keratinocytes to support the immune system, which contributes to inflammation, and thus further promote the pathogenesis of psoriasis (Takahashi and Yamasaki, 2020). In contrast to TH17 cytokines, TH2 cytokines are thought to inhibit AMP expression in patients with AD (Nomura et al., 2003; Ong et al., 2002). This, in turn, might be associated with a higher predisposition to *Staphylococcus aureus* colonization in AD, as patients with AD are often pathologically colonized with *S. aureus* (Abeck and Mempel, 1998).

A simple method to mimic an inflammatory skin disease phenotype *in vitro* is to add disease-relevant cytokines to 3D skin models. AD models can be generated by adding TH2 cytokines such as IL-4 and IL-13, and TNF-α (Danso et al., 2014; De Vuyst et al., 2018; Kamsteeg et al., 2011), whereas psoriasis models are induced by treating skin models with cytokine cocktails based on IL-17, IL-22 and TNF-α. Integration of disease-relevant immune cells, such as CD4^+^ T cells, TH1 or TH17 cells, into skin model can also induce an inflammatory skin disease phenotype (Moon et al., 2021; Shin et al., 2020; van den Bogaard et al., 2014). It is important that models of skin diseases physiologically recapitulate skin disease to be an effective platform for study of the disease. In this study, we thoroughly analyzed psoriasis and AD skin models induced by cytokine stimulation or by integrated TH1 CD4^+^ T cells for histology and function. Here, we demonstrate that we successfully developed 3D human psoriasis and AD skin models exhibiting several clinical features of the corresponding human inflammatory skin diseases.

## RESULTS

### Establishment of full-thickness 3D skin equivalents that closely resemble human skin

**Fig. 1.**
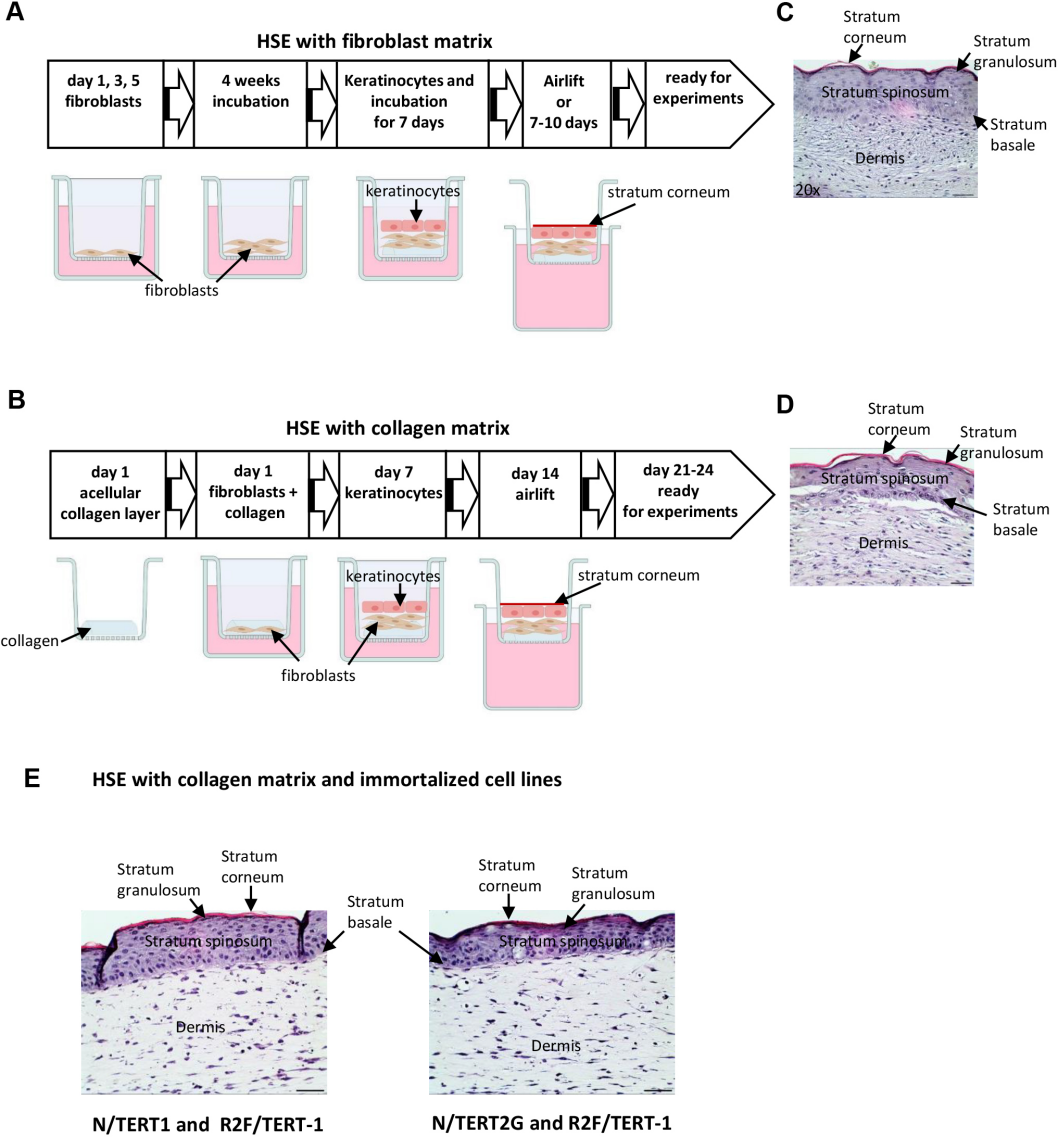
**Establishment of 3D human skin equivalents.** (A,B) Flowchart of human skin equivalent (HSE) generation using a fibroblast matrix (A) or a collagen matrix (B). Figure created with Biorender.com. (C,D) Histological sections of HSE with primary human cells were stained with Hematoxylin and Eosin (H&E). The image shown in C is taken from the experiment shown in Fig. 2A. (E) HSEs based on a collagen matrix were generated using the immortalized keratinocyte cell line N/TERT1 or N/TERT2G and the fibroblast cell line R2F/TERT-1. Shown are histological sections of HSEs stained with H&E. Scale bars: 100 µm (C-E).

We developed 3D HSEs built up on either a fibroblast matrix or a collagen matrix. For the generation of HSEs based on a fibroblast matrix, we modified a protocol described by Berning et al. (2015). Fibroblasts were seeded directly onto transwell inserts three times (days 1, 3 and 5). The dermal compartment was then cultured for at least 4 weeks before keratinocytes were seeded onto it. After an additional 7 days of culture, the tray was lifted and the top was exposed to air, which induces keratinocyte differentiation and stratum corneum formation (Fig. 1A). For the generation of HSEs based on a collagen matrix, fibroblasts embedded in collagen were seeded on collagen-coated transwell inserts. After the fibroblasts had constricted the collagen (7 days after fibroblast seeding), keratinocytes were seeded on top of the dermal compartment. After another 7 days of culture, the tray was lifted and the top exposed to air for the formation of the stratum corneum (Fig. 1B). HSEs with a fibroblast matrix have a longer manufacturing time, but they have the advantage of a longer lifespan of up to 3 months (Berning et al., 2015), and were therefore mainly used for studies with longer analysis times. Although it is more timesaving to prepare HSEs based on a collagen matrix, they have a short lifetime of up to 2 weeks, and were thus used especially for experiments with short analysis times. Independent of the approach used for HSE generation, the tissue structure was well developed and very similar to human skin. The HSEs consisted of a dermis and a fully differentiated epidermis with hierarchically arranged epidermal layers: stratum basale, stratum spinosum, stratum granulosum and stratum corneum (Fig. 1C,D). High expression of the basement membrane protein type IV collagen further indicated that an intact basement membrane separating the epidermis from the dermis was formed with proliferating cells at the junction zone as seen by Ki-67 staining (Fig. S1A,B).

**Fig. 2.**
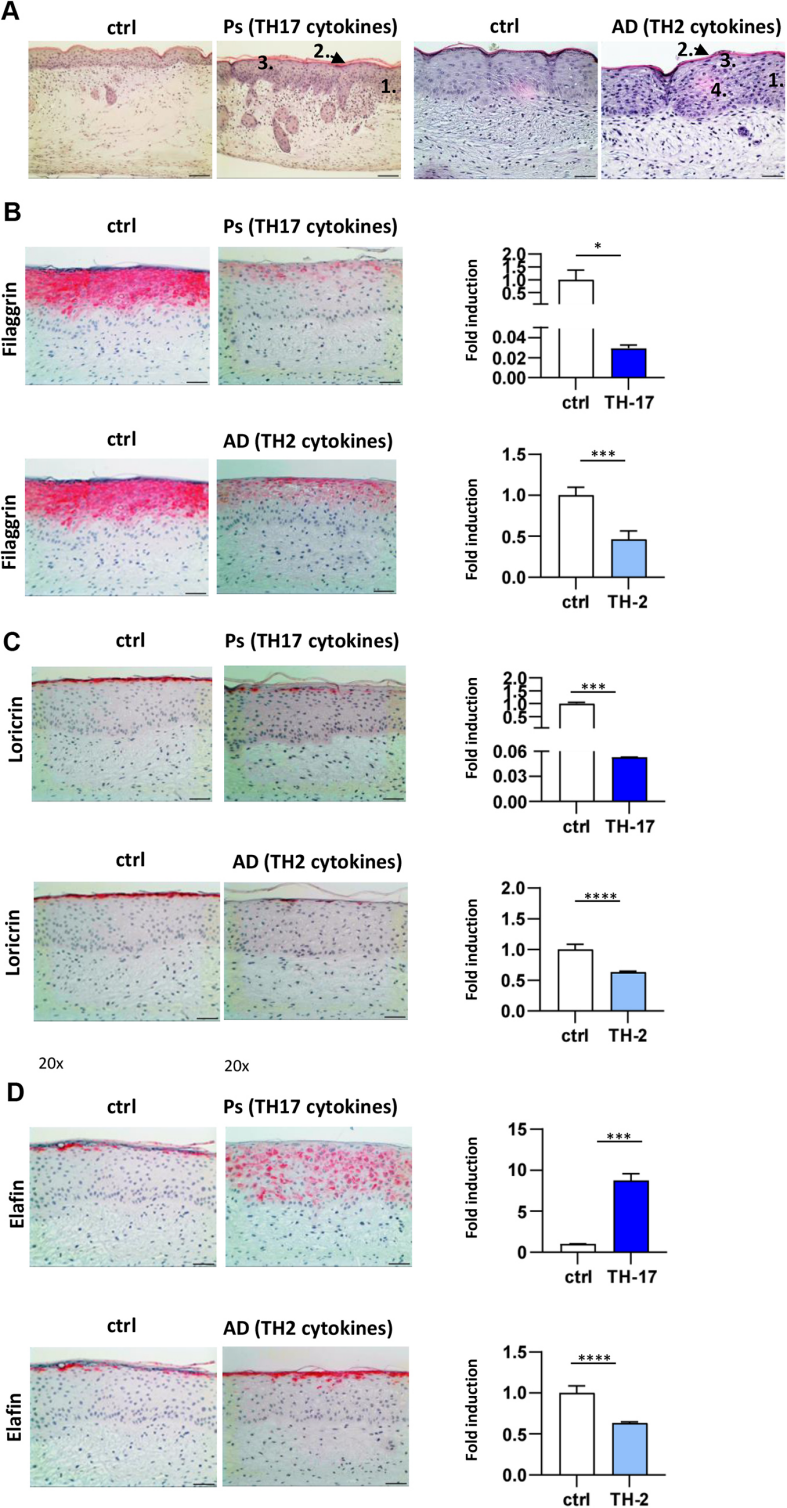
**Cytokine-induced skin models of atopic dermatitis and psoriasis.** (A) Histological sections of control, TH17-induced psoriasis (Ps) or TH2-induced atopic dermatitis (AD) HSEs based on a fibroblast matrix were stained with H&E. Disease induction is manifested by epidermal thickening (1), parakeratosis (2), hypogranulosis (3) and spongiosis (4) in the inflammatory skin constructs. The AD control HSE image is also shown as an illustrative example in Fig. 1C. (B-D) Immunohistology of control, psoriasis and AD models was performed for filaggrin (B), loricrin (C) and elafin (D). Cytokine treatments to generate psoriasis and AD HSEs were performed simultaneously as part of the same experiment using a shared control, which is shown alongside both the psoriasis and AD HSE in each panel for direct comparison. Images are representative of at least three independent experiments. Scale bars: 100 µm (A-D). Additionally, fold induction of gene expression of the respective markers was determined for each model (*n*=2-4 technical replicates). Error bars show s.d. Statistical analysis was performed with an unpaired two-tailed parametric *t*-test. *P<0.05; ***P<0.001; ****P<0.0001.

We also used immortalized human cell lines for collagen-based HSE generation: the immortalized human keratinocyte cell lines N/TERT1 or N/TERT2G (Dickson et al., 2000), and the human fibroblast cell line R2F/TERT (Dickson et al., 2000) (Fig. 1E). When immortalized keratinocyte and fibroblast cell lines were used, a good tissue structure was developed that closely resembled human skin. The advantages of constructing HSEs with cell lines instead of primary cells are that they have an unlimited lifespan and are suitable for genome-editing techniques. Therefore, these cell lines might be an important tool for studies on genetic risk factors for skin diseases.

### Cytokine-induced skin models of AD and psoriasis exhibit phenotypic hallmarks of the corresponding diseases

An inflammatory skin disease phenotype was induced by 3-5 days of cytokine stimulation of the fibroblast matrix-based HSE during the airlift phase. In the case of the psoriasis model, a TH17-associated cytokine cocktail (IL-17A and IL-22) was used, and in the case of the AD model, a TH2-associated cytokine cocktail (IL-4, IL-5 and IL-13) was used. A major consequence of the cytokine-rich environment in psoriasis and AD is disturbed keratinocyte differentiation within the lesions (Zhou et al., 2022). We observed that epidermal keratinocytes responded to the addition of TH17- and TH2-associated cytokines with hyperproliferation and abnormal and dysfunctional differentiation. This was manifested by epidermal thickening (1), parakeratosis (2), hypogranulosis (3) and spongiosis (4) in the skin constructs (Fig. 2A), which are changes that are also commonly observed in patients. In addition, we investigated the induction of a disease-like profile by examining molecular markers known to be altered in expression in keratinocytes in psoriasis and AD skin. As expected in an inflammatory skin disease, both AD and psoriasis models showed disturbed epidermal differentiation with decreased protein and mRNA expression of the late keratinocyte differentiation markers filaggrin (FLG) (Fig. 2B) and loricrin (Fig. 2C). In contrast, increased protein and mRNA expression of the early differentiation marker elafin (PI3) was detectable in the spinous layer of the psoriasis model, whereas in the AD model, elafin expression was only increased at the protein level (Fig. 2D). Overall, these data indicate the psoriasis and AD models mimic the human inflammatory skin disease phenotypes very well.

### Multiplexed imaging validates phenotypic hallmark expression in 3D skin models

**Fig. 3.**
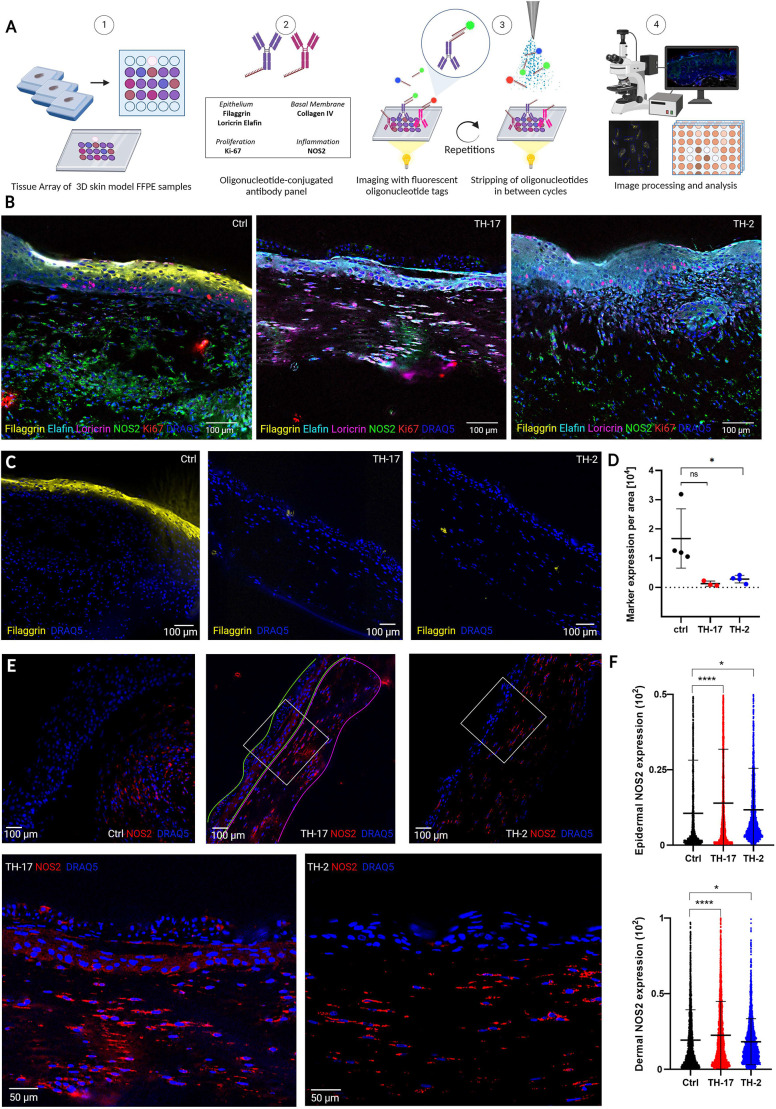
**Analysis of 3D human skin models by highly multiplexed imaging (CODEX).** (A) CODEX workflow from 3D skin model to processed image. FFPE, formalin-fixed paraffin-embedded. Figure created with Biorender.com. (B) Overlay of representative 3D human skin models, showing the epithelial markers filaggrin, loricrin and elafin, Ki-67, NOS2 and the nuclear stain DRAQ5. Left, untreated HSEs in six-well collagen matrix; middle, HSEs treated with TH17 cytokines for 3 days in six-well collagen matrix; right, HSEs treated with TH2 cytokines for 3 days in six-well collagen matrix. Images are representative of at least three independent experiments. (C-F) Shown is either the expression or expression level of single markers using the same HSEs as shown in B and in Figs S1 and S2. (C) CODEX fluorescence image comparison of filaggrin expression in untreated control, TH17 and TH2 3D human skin models. 3 day cytokine treatment, six-well collagen matrix. (D) Comparison and unpaired two-tailed *t*-test of mean filaggrin expression per unit area in the epidermis of all analyzed 3D human skin models (*n*=4). (E) CODEX fluorescence image comparison of NOS2 expression in untreated control, TH17 and TH2 3D human skin models. 3 day cytokine treatment, six-well collagen matrix. The outlines (top) show regions of interest for statistical analysis (green, epidermis; magenta, dermis). Bottom: magnification of results for TH17- and TH2-treated models showing epidermal NOS2 expression patterns in the psoriasis model. (F) Statistical comparison of NOS2 expression per epithelial (top) or dermal (bottom) cell by unpaired two-tailed *t*-test. In D,F, bars represent the mean±s.d. Scale bars: 100 μm (B,C and upper panels in E); 50 µm (E, lower panels). ns, not significant; *P<0.05; ****P<0.0001.

‘Co-detection by indexing’ (CODEX) is a highly multiplexed microscopy technology based on iterative annealing and chemical stripping of fluorescently labeled DNA probes that bind to DNA-conjugated antibodies (Black et al., 2021; Kennedy-Darling et al., 2021). CODEX enables tissue imaging with all chosen parameters simultaneously per sample (Fig. 3A). Imaging with CODEX was performed to validate the expression analyses done by immunohistology (Fig. 3A). For this purpose, the best epidermal regions from paraffin blocks of collagen-based HSEs were selected for preparation of a tissue array, including five samples of untreated skin models, five samples of cytokine models for the AD phenotype and five skin models for the psoriasis phenotype. For CODEX analysis, DNA-conjugated antibodies were used to detect epithelial, activation/proliferation and inflammation markers. We performed CODEX analysis for the markers filaggrin, elafin, loricrin, Ki-67 and inducible nitric oxide synthase (NOS2) of the TH17-induced psoriasis model (‘TH17’) and TH2-induced AD model (‘TH2’) and compared the results to the untreated control group (Fig. 3B). The combined CODEX analysis of all analyzed markers is shown in Fig. 3B for an untreated control model and representative cytokine models for the AD and psoriasis phenotypes. In Fig. 3C-F and Fig. S2A-D, we show individual marker expression and quantitative analyses between the samples. The CODEX analysis in principle validated our results from the immunohistological staining. We found a strong downregulation of filaggrin expression in the AD and psoriasis models compared to that in the untreated control models (Fig. 3C,D), as well as changes in the expression of loricrin and elafin in the psoriasis and AD models (Fig. S2A-D). Garzorz-Stark et al. (2016) described that NOS2 is significantly upregulated in lesional psoriatic skin compared to in healthy skin or AD. Indeed, we found that NOS2 is expressed more prominently and is more widely spread in the epidermis of the psoriasis model compared to the epidermis of the control HSE and the AD model (Fig. 3E,F).

### Establishment of an immunocompetent psoriasis skin model using integrated TH1 cells

**Fig. 4.**
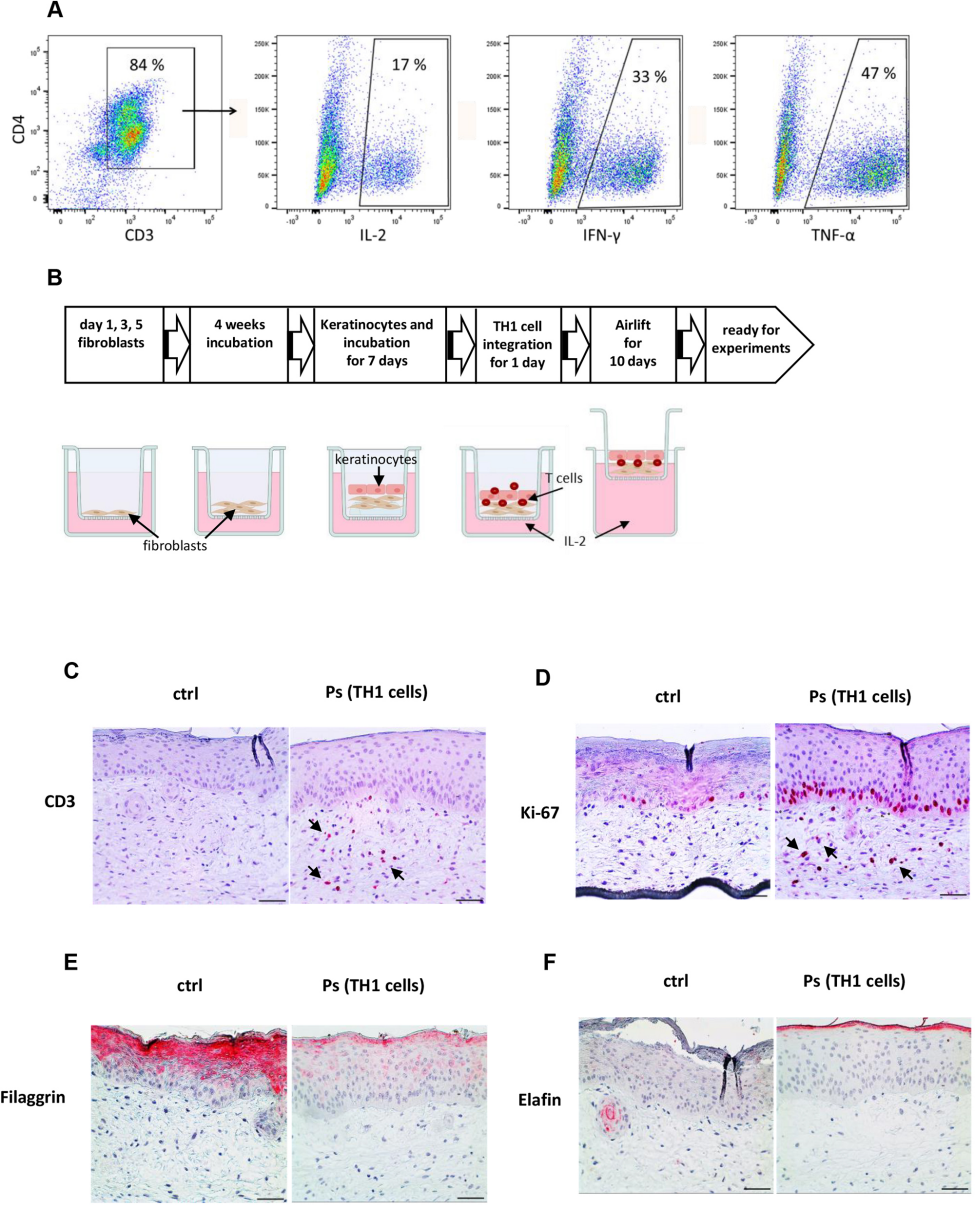
**Immunocompetent 3D psoriasis model.** (A) Flow cytometry data of the cytokine profile of TH1-polarized CD4^+^ T cells 2 weeks after stimulation. We stimulated human CD4^+^ T cells isolated from peripheral blood mononuclear cells with Dynabeads Human T-Activator CD3/CD28 and a TH1 cytokine cocktail (10 ng/ml IFN-γ, 10 ng/ml IL-12 and 1 µg/ml anti-IL-4) for 2 weeks. (B) Flowchart of HSE generation using a fibroblast matrix and T cell integration. Figure created with Biorender.com. (C-F) Histological sections of an immunocompetent psoriasis HSE based on a fibroblast matrix and integrated TH1-polarized T cells were stained with antibodies against CD3 to detect integrated TH1 cells in the dermis (C; red staining; examples marked with arrows), the proliferation marker Ki-67 (D; red staining detects T cells in the dermis and cells in the basal epidermal layer at the basement membrane; examples marked with arrows), filaggrin (E) and elafin (F). ctrl, untreated HSE; Ps, HSE with integrated TH1 cells. Images are representative of at least three independent experiments. Scale bars: 100 µm (C-F).

The use of immunocompetent skin models to study immune cell–skin cell interactions and the role of immune cells in inflammatory skin diseases is of great interest. Therefore, we developed a 3D psoriasis model with integrated TH1-polarized CD4^+^ T cells. We stimulated human CD4^+^ T cells isolated from peripheral blood mononuclear cells with a TH1 cytokine cocktail (10 ng/ml IFN-γ, 10 ng/ml IL-12 and 1 µg/ml anti-IL-4) for 2 weeks. *In vitro*-polarized TH1 T cells (Fig. 4A) were integrated in fibroblast matrix-based HSEs 1 day prior to performing the airlift phase, and the HSEs were then analyzed 10 days after the airlift phase (Fig. 4B). During this time, 10 ng/ml IL-2 was added to maintain T cell viability in the skin model. Interestingly, the TH1-polarized T cells alone could induce a psoriatic phenotype as seen by thickening of the epidermis and parakeratosis (Fig. 4C). Integrated TH1 cells were detectable in the dermal compartment – the natural habitat of activated T cells – by CD3 staining (Fig. 4C) and were viable as detected by Ki-67 staining (Fig. 4D). Interestingly, integrated TH1 cells were able to change the expression of epidermal barrier proteins as seen in patients with psoriasis, as evidenced by reduced expression of filaggrin (Fig. 4E) and increased expression of elafin (Fig. 4F).

### Multiphoton microscopy of 3D skin equivalents reveals a correct spatial arrangement of the epidermal layers

**Fig. 5.**
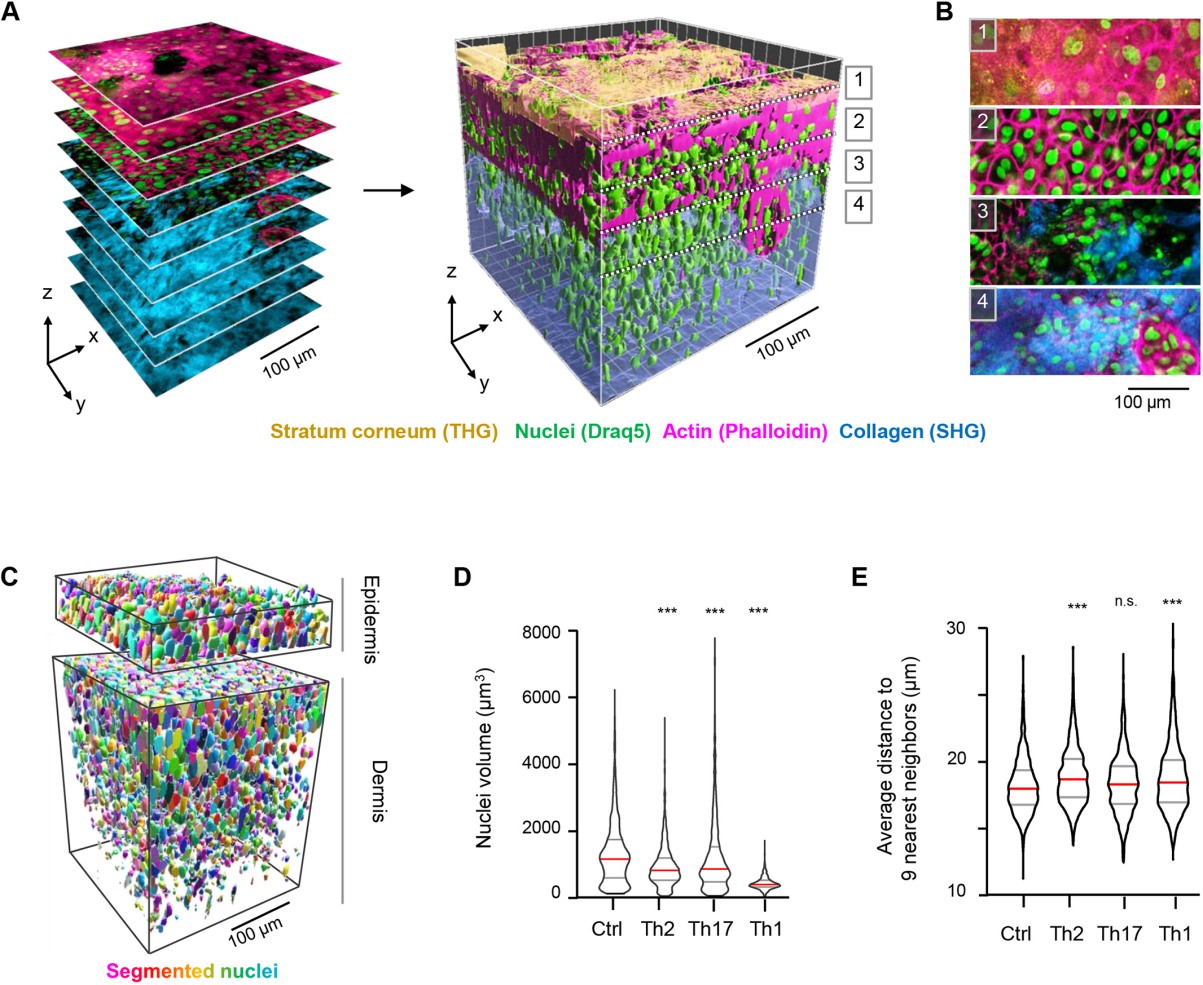
**Multiphoton microscopy of 3D skin equivalents.** Multiphoton 3D reconstruction and quantification of collagen-based HSEs. (A) Image stacks (left) were taken at 1 μm *z*-steps and reconstructed to a 3D volume (right) for surface rendering of nuclei (green, nuclei surface based on DRAQ5 labeling and rendering; magenta, keratinocytes in the epidermal layer, phalloidin–Alexa Fluor 546; yellow, stratum corneum, third harmonic generation; cyan, collagen fibers, second harmonic generation). (B) Zoomed images at higher resolution taken from the slices indicated in A. (C) Segmentation of nuclei in epidermal and dermal layers. The epidermal layer was defined based on the phalloidin signal, and the dermal layer based on the presence of collagen. (D) Quantification of nuclei volumes in the epidermis in the distinct disease conditions. (E) Quantification of nuclei density plotted as the average distance of each nucleus to the nine nearest neighboring nuclei. For D,E, 1000 nuclei were analyzed per condition from one independent experiment. In violin plots, red line indicates the median, grey lines indicate the 25-75th percentiles. ***P<0.0001 (Kruskal–Wallis test).

To further characterize HSEs using primary human cells in more detail, we performed multiphoton microscopy of an HSE based on a collagen matrix. HSE whole-mount staining of nuclei (detected by DRAQ5) and actin filaments (detected by fluorescently-labeled phalloidin), followed by 3D reconstruction using near-infrared multiphoton excitation and second and third harmonic generation (SHG/THG) further confirmed the generation of several differentiated epidermal layers (Fig. 5A,B). THG visualizes interfaces of materials with a distinct refractive index and, in HSEs, THG signals originated predominantly from the stratum corneum and granulosum, comparable to what is seen for native human skin (Weng et al., 2016). SHG contrast marked the collagen-rich dermal compartment of the HSEs, and phalloidin labeled the keratin filaments in keratinocytes and allowed visualization of their morphology in distinct differentiation states and epidermal layers (Fig. 5B).

AD and psoriasis HSEs were further 3D reconstructed by multiphoton microscopy in 350×350×800 µm volumes and keratinocyte nuclei were segmented and volume-rendered (Fig. 5C). This allowed quantification of nuclear size and density variations between conditions and confirmed disease-specific morphological changes, such as nuclear condensation (Fig. 5D) and spongiosis, characterized by larger distances between individual nuclei (Fig. 5E).

### Psoriasis and AD skin disease models resemble clinical phenotypes in terms of AMP production and *S. aureus* colonization

**Fig. 6.**
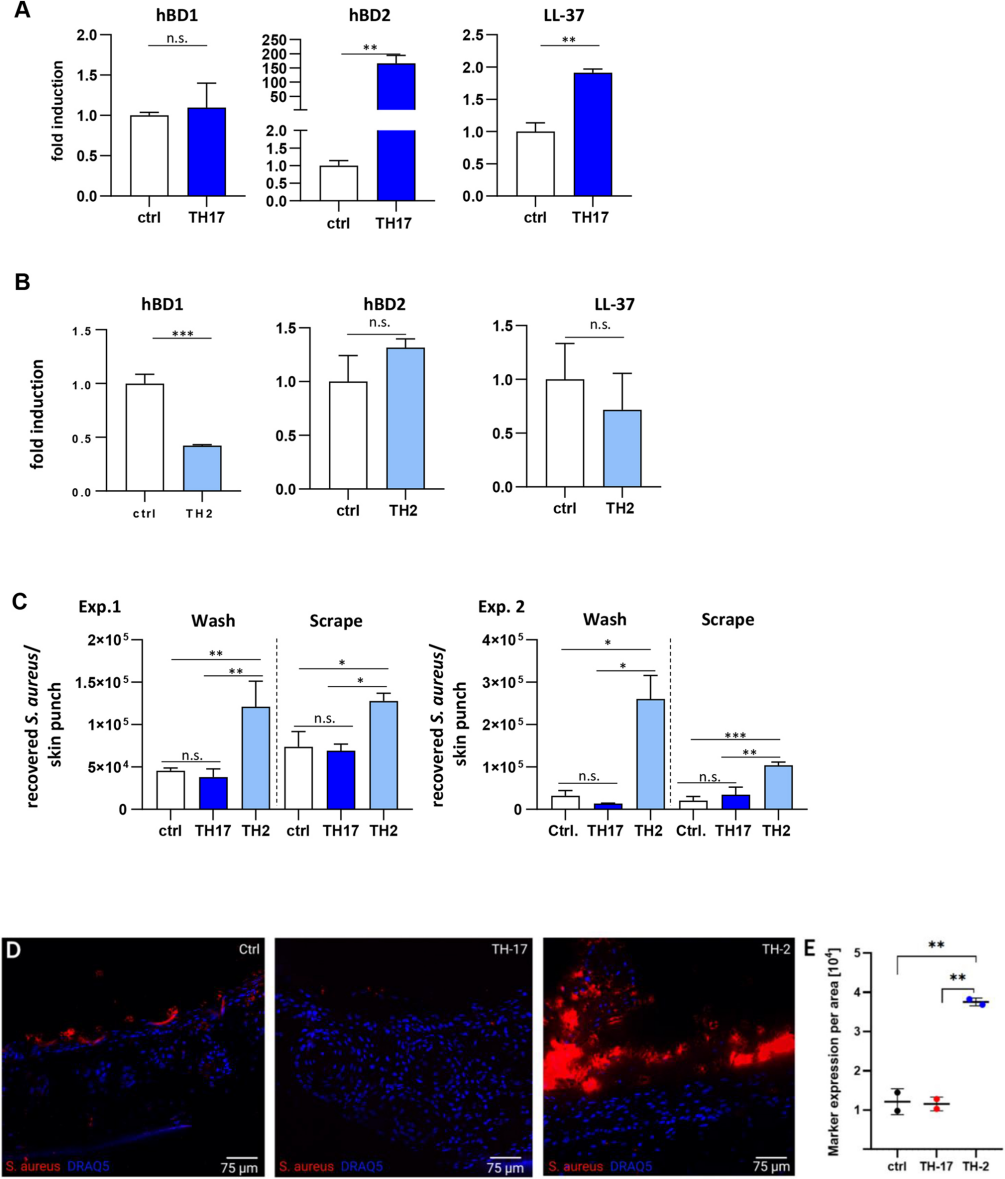
**Antimicrobial peptide production and *S. aureus* colonization in psoriasis and AD models.** (A,B) Fold induction of gene expression of the antimicrobial peptides hBD1, hBD2 and LL-37 in collagen-based psoriasis models (A) and AD models (B) was analyzed by qRT-PCR and compared to gene expression in untreated control HSE (collagen-based HSE) (*n*=2-4 technical replicates). Unpaired two-tailed parametric *t*-test was used. (C) Collagen-based HSEs were infected with *S. aureus* and a colony-formation assay was performed. Thereafter, HSEs were washed and homogenized, and the wash solution (to quantify loosely attached *S. aureus*; labeled ‘wash’) and the homogenate (to quantify infiltrated bacteria; labeled ‘scrape’) were plated onto blood agar plates. Two independent experiments with *n*=2-3 technical triplicates are shown. One-way ANOVA followed by Dunnett's multiple comparisons test was used. ctrl, untreated HSEs; TH17, psoriasis model induced by TH17 cytokine stimulation; TH2, AD model induced by TH2 cytokine stimulation. (D) CODEX fluorescence image comparison of *S. aureus* expression in *S. aureus*-infected untreated control, *S. aureus-*infected TH17 and *S. aureus*-infected TH2 3D human skin models. 3 day cytokine treatment, six-well collagen matrix. Scale bars: 75 μm. (E) Comparison and unpaired two-tailed *t*-test of mean marker expression of *S. aureus* per area in the epidermis of infected 3D human skin models (*n*=2). Unpaired two-tailed *t*-test was used. n.s., not significant; *P<0.05; **P<0.01; ***P<0.001.

Cytokines including IL-17 and IL-22 have been described to promote AMP production by keratinocytes. We demonstrated that mRNA expression of hBD2 (*DEFB4A*) and LL-37 was significantly increased in TH17 cytokine-induced collagen-based psoriasis models compared with expression in untreated skin constructs, but expression of hBD1 (*DEFB1*) was not enhanced (Fig. 6A). Therefore, AMP production in psoriasis models is comparable to that observed in patients with psoriasis (Takahashi and Yamasaki, 2020). In contrast to TH17 cytokines, TH2 cytokines are thought to inhibit AMP expression. This, in turn, is associated with a higher predisposition to *S. aureus* colonization in AD. Treatment with TH2 cytokines (IL-4, IL-5 and IL-13) significantly decreased hBD1 mRNA expression in the collagen-based AD model, whereas the expression of other AMPs analyzed, including hBD2 and LL-37, was not significantly affected (Fig. 6B). Furthermore, we investigated the ability of *S. aureus* to colonize the control HSE and the TH2 or TH17 cytokine-treated collagen-based skin models by performing a colony-formation assay. Therefore, *S. aureus*-infected HSEs were washed and then homogenized, and the wash solution (to quantify loosely attached *S. aureus*; labeled ‘wash’) and the homogenate (to quantify infiltrated bacteria; labeled ‘scrape’) were plated onto blood agar plates. Compared to untreated control or psoriasis models, only the AD models showed enhanced colonization (‘wash’) and infiltration (‘scrape’) with *S. aureus* (Fig. 6C), possibly due to decreased AMP production. Those results were validated by CODEX analysis, using which we showed a strong colonization in infected AD model, distinguishing it from low amounts of *S. aureus* expression in the control and even lower expression in the psoriasis model (Fig. 6D,E).

**Fig. 7.**
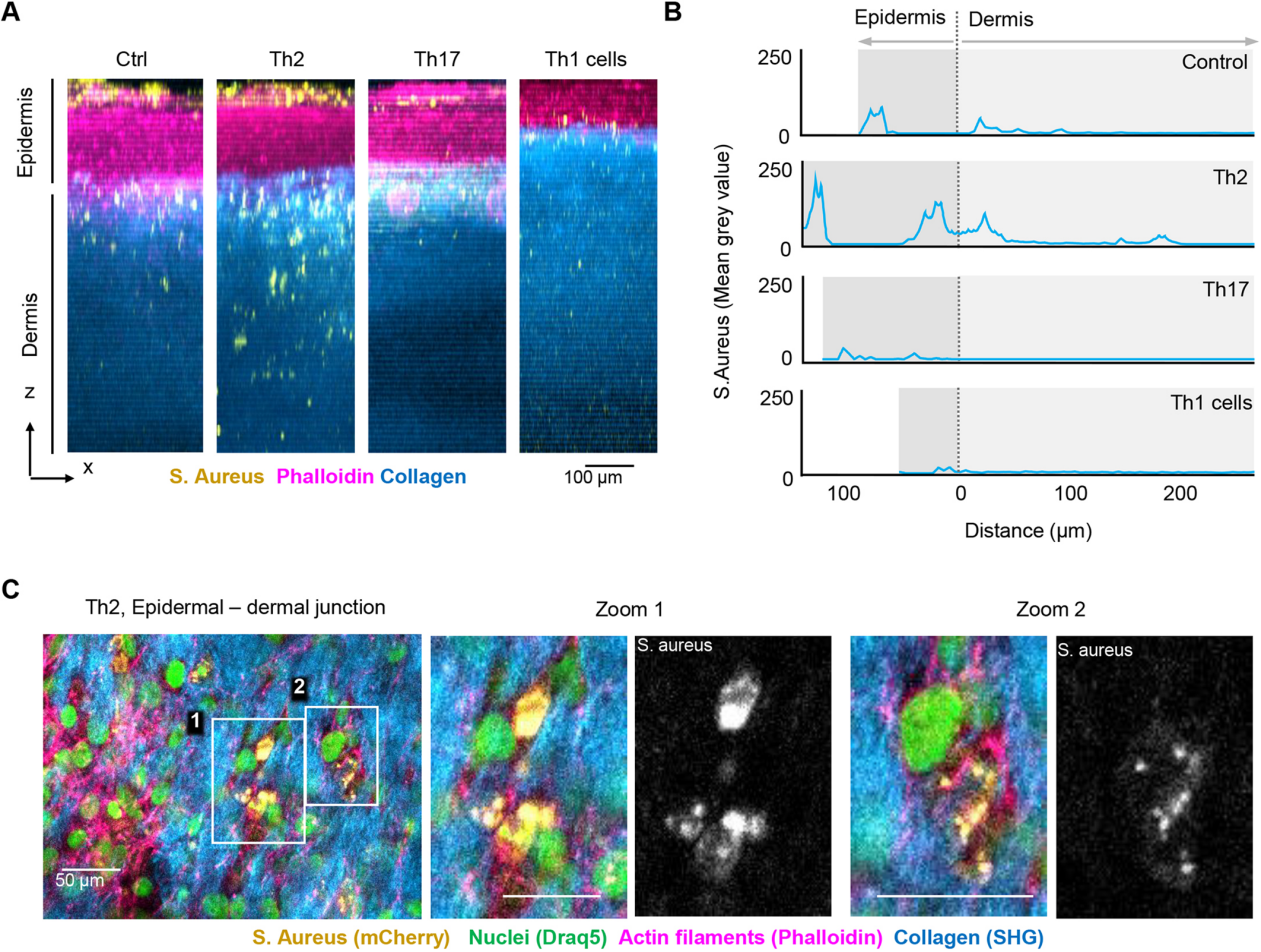
**Multiphoton 3D reconstruction of *S. aureus* colonization and infiltration depth.** (A) Maximum projections of 80 *zx* slices derived from 350×350×800 μm volumes of untreated collagen-based control, TH2-induced AD, and TH17 cytokine- and TH1 T cell-induced fibroblast matrix-based psoriasis models. Scale bar: 100 μm. (B) Epidermal and dermal layers were defined based on the phalloidin and collagen signals and mCherry-expressing *S. aureus* density was quantified based on the mCherry signal intensity (mean gray value). (C) High-resolution image and zoomed views of *S. aureus* colonization of the epidermal–dermal junction in the TH2-induced AD model. Yellow, *S. aureus* (mCherry); green, nuclei (DRAQ5); magenta, actin filaments (phalloidin–Alexa Fluor 546); blue, collagen fibers (second harmonic generation). Scale bars: 50 μm. Images are representative of one experiment.

We further quantified the infiltration depth of mCherry-expressing *S. aureus* in the collagen-based HSEs using multiphoton 3D volume reconstruction (Fig. 7A). Compared to the untreated control, *S. aureus* showed increased colonization rates and infiltration depth in the AD model, with an accumulation at the stratum corneum and the epidermal–dermal junction (Fig. 7B). In both TH17 cytokine- and TH1 T cell-induced psoriasis models, *S. aureus* growth was strongly reduced (Fig. 7B). High-resolution multiphoton imaging in whole mounts showed that *S. aureus* formed small colonies in the stratum basale and interfacing dermal layers and was often found in close contact with keratinocytes (Fig. 7C). Overall, these data show that the psoriasis and AD models differ in this regard at the functional level and correlate well with the clinical phenotypes in respective patients.

### JAK1/3 inhibition normalizes skin disease phenotypes in the inflammatory skin models

**Fig. 8.**
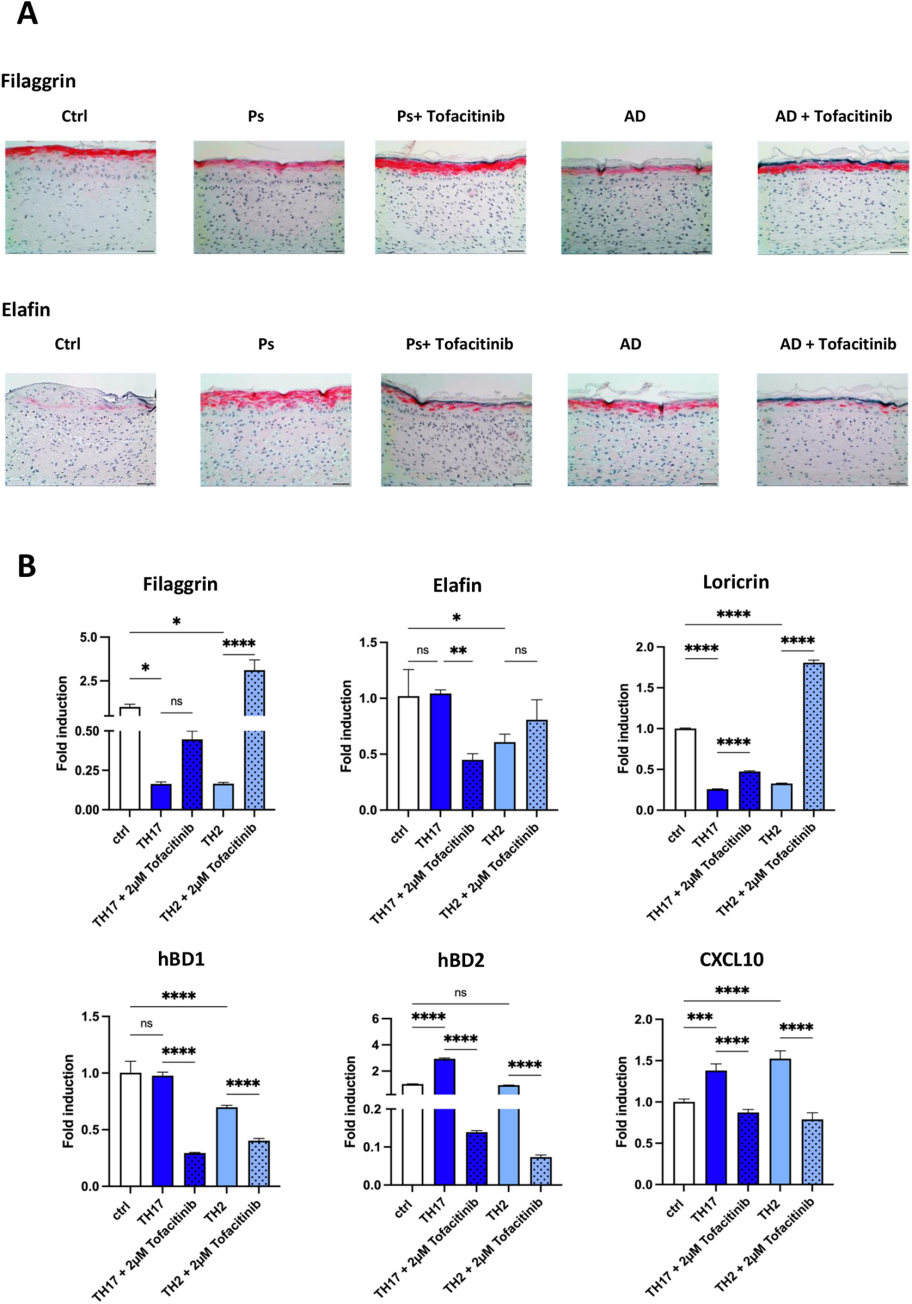
**JAK1/3 inhibition normalizes skin disease phenotype.** (A) Histological sections of tofacitinib-treated (2 µM) TH17-induced psoriasis, TH2-induced AD or control HSEs based on a fibroblast matrix. Immunohistology of control, psoriasis and AD models was performed for filaggrin and elafin. Images are representative of at least three independent experiments. Scale bars: 100 µm. (B) Additionally, fold induction of gene expression of the indicated markers was determined by qRT-PCR for each model (*n*=2-4 technical replicates). Statistical analysis was performed by one-way ANOVA followed by Dunnett's multiple comparisons test. ns, not significant; *P<0.05; **P<0.01; ***P<0.001; ****P<0.0001.

Because psoriasis and AD models exhibit disease morphology as observed in patients, they could serve as an attractive platform for testing new treatment strategies. We therefore asked whether these models can be reliably used for drug treatment research. For this purpose, we investigated the effect of tofacitinib (JAK1/3 inhibitor) on disease phenotype improvement in the fibroblast-matrix based AD and psoriasis models. To assess the effects of the drug on improving disease phenotype, we examined the expression of the epithelial markers filaggrin, loricrin and elafin in the epidermis of cytokine-induced psoriasis models and AD models before and after tofacitinib treatment by immunohistology (Fig. 8). We observed that JAK1/3 inhibition led to normalization of mRNA and protein expression of all analyzed markers and improved the overall skin morphology (Fig. 8A,B). Furthermore, JAK1/3 inhibition reduced expression of the AMPs hBD1 and hBD2 and of the proinflammatory cytokine CXCL10 (Fig. 8B). These data indicate that the established psoriasis and AD models can be used to test novel treatment strategies.

## DISCUSSION

3D human skin models are an attractive tool to study human skin, especially because mouse models often do not translate the human system owing to differences in skin structure and immune cell composition (Mestas and Hughes, 2004; Pasparakis et al., 2014; Salgado et al., 2017). At the same time, it is difficult to obtain enough sample material from patient biopsies and to keep them viable long enough for research (Todorovic et al., 2022). However, when using 3D skin models, it is important that they accurately reproduce the human skin or the skin disease under investigation physiologically and pathologically in order to be an effective platform for studies. With our study, we provide detailed profile characterization of models of the inflammatory skin diseases psoriasis and AD, induced by either cytokine treatment or TH1-polarized T cell integration, using immunohistochemistry, multiplex microscopy, multiphoton microscopy and mRNA expression analysis.

For our studies, we used HSE models in which the dermis was either created with fibroblasts embedded in a collagen I matrix (collagen-based model) or in which the dermal compartment was created by direct seeding of fibroblasts onto the transwell matrix (fibroblast matrix model). In both models, the epidermis was constructed with stratified, differentiated keratinocytes. HSEs with the fibroblast matrix are more time-consuming to prepare and have a longer manufacturing time. However, they have the advantage of a longer lifetime of up to 3 months (Berning et al., 2015) and are therefore particularly suitable for long-term studies, whereas HSEs produced on a collagen matrix only have a lifetime of up to 2 weeks. Both approaches produce tissue with correct skin morphology and barrier function and consist of a dermis and a fully differentiated epidermis with hierarchically arranged epidermal layers, namely, stratum basale, stratum spinosum, stratum granulosum and stratum corneum. Cell-to-cell and cell-to-extracellular matrix interactions within the 3D model are important here for physiologically relevant cell functions that cannot be achieved with a two-dimensional (2D) cell culture with human keratinocytes, fibroblasts and immune cells. Additionally, 2D cell cultures lack the environmental factors such as mechanical forces, spatial orientation, and physiological oxygen, nutrient and signaling gradients that occur in the 3D *in vivo* environment (Randall et al., 2018).

As TH17 cells have been described as main contributors for the development of psoriasis pathology (Kryczek et al., 2008; Lowes et al., 2008), a psoriasis model was induced by treating HSEs with a TH17-derived cytokine mix (IL-17A and IL-22, 30 ng/ml each). To induce an AD phenotype, HSEs were treated with a TH2-associated cytokine mix (20 ng/ml IL-4, 10 ng/ml IL-5 and 10 ng/ml IL-13), as AD is mainly a TH2-driven disease (Fiset et al., 2006). The psoriasis and AD skin disease models showed abnormal and dysfunctional epidermal differentiation as observed in inflammatory skin disease *in vivo*. This included the development of a hyperplastic and thickened epidermis, with signs of parakeratosis, hypogranulosis and spongiosis. In addition, impaired epidermal differentiation is associated with dysregulated expression of molecular markers, as evidenced by decreased expression of the late keratinocyte differentiation marker filaggrin and loricrin, and increased expression of the early differentiation marker elafin. Although the pathological immunoreaction in psoriasis and AD is opposite, both have similar phenotypic features in terms of changes in epidermal differentiation and the epidermal compartment, which we also observed in our 3D skin disease models. To distinguish inflammatory skin diseases at a functional level, we analyzed the induction of AMPs and the ability of *S. aureus* to colonize skin models. It has been described that the psoriasis-associated cytokines IL-17 and IL-22 promote the secretion of AMPs such as hBD2 and LL-37 by keratinocytes (Takahashi and Yamasaki, 2020). Although AMPs mainly act as natural antibiotics, in the case of psoriasis, they stimulate the immune system, further promoting inflammation and pathogenesis of psoriasis (Takahashi and Yamasaki, 2020). In contrast to TH17 cytokines, TH2 cytokines are thought to inhibit AMP expression in patients with AD (Nomura et al., 2003; Ong et al., 2002), which in turn may be associated with increased *S. aureus* colonization in AD, as patients with AD are often pathologically colonized with *S. aureus* (Abeck and Mempel, 1998). We observed that the expression of the AMPs hBD2 and LL-37 was significantly increased in TH17 cytokine-induced psoriasis models compared with that in untreated skin constructs. In contrast, treatment with TH2 cytokines significantly decreased hBD1 expression in the AD model. In relation to reduced AMP expression in the AD model, we also demonstrated that the AD model exhibited increased colonization and infiltration with *S. aureus*, compared to untreated HSE or the psoriasis model. These data demonstrate that the psoriasis and AD skin models not only mimic psoriasis and AD well histologically, but also correlate well with clinical phenotypes at the functional level.

In addition to the use of cytokines, HSEs with integrated CD4^+^ T cells or TH1- and TH17-polarized T cells have already been developed to study the pathogenesis of psoriasis (Moon et al., 2021; Niehues and van den Bogaard, 2018; Stanton et al., 2022; van den Bogaard et al., 2014). The reactivity of the immune cells integrated into HSEs has been described to be comparable to that *in vivo* (Facy et al., 2005; Niehues et al., 2018). This shows the potential and clinical relevance of the use of especially immunocompetent 3D skin models (Moon et al., 2021; Stanton et al., 2022). Moreover, these models can be useful for the investigation of immune cells or immune cell–skin cell interactions in inflammatory skin diseases. Here, we established a psoriasis model with TH1-polarized T cells integrated into HSEs one day before performing the airlift phase. The reactivity of the integrated TH1 T cells in the HSEs is comparable to that *in vivo*, as these HSEs developed an inflammatory skin disease phenotype with impaired epidermal differentiation associated with reduced filaggrin expression and increased elafin expression.

As psoriasis and AD models exhibit disease morphology as observed in patients, they could be an attractive platform for new drug discovery. Therefore, we used these models to test the effect of potential drug treatment. For our studies, we chose tofacitinib (JAK1/3 inhibitor), which is an attractive therapeutic agent for improving the phenotype of psoriasis and AD. Tofacitinib inhibits intracellular JAK/signal transducers and activators of transcription (STAT) signaling pathways, both of which play a role in pro-inflammatory effects of many cytokines that are also present in AD and psoriasis (Clarysse et al., 2019; O'Shea and Murray, 2008; O'Shea et al., 2015). Clinical trials have already shown that the application of tofacitinib can improve psoriasis (Papp et al., 2016) and eczema areas (Bissonnette et al., 2016). In our studies we showed that the pharmacological effects in HSEs obtained with tofacitinib correlate with therapeutic effects observed in the clinic. JAK1/3 inhibition had a profound effect on normalizing skin morphology as well as expression of markers of skin differentiation, AMPs and proinflammatory cytokines in both psoriasis and AD models.

In summary, by performing an in-depth analysis using immunohistochemistry, multiplex microscopy, 3D multiphoton whole-mount reconstruction and mRNA expression analysis, our study demonstrates that psoriasis and AD models exhibit several clinical features of inflammatory diseases, thus providing a very good platform to study inflammatory skin diseases. Therefore, it is very important to consider the benefit of 3D skin models in experimental dermatological research and in the assessment of new drugs.

## MATERIALS AND METHODS

### Cell culture

Primary human fibroblasts and keratinocytes were isolated from human infantile foreskin after routine circumcision as previously described (Bitschar et al., 2019; Burian et al., 2017). Fibroblasts were cultured in tissue flasks in CnT-Prime Fibroblast Proliferation Medium (CELLnTEC) at 37°C and 5% CO_2_. Keratinocytes were cultured in collagen-coated tissue flasks in CnT-BM.1 Basal Medium 1 with supplements (CELLnTEC) at 37°C and 5% CO_2_. 2-3 days before using the keratinocytes for experiments, keratinocytes were cultured in CnT-Prime Epithelial Proliferation Medium (CELLnTEC). For experiments, cell passages 2-4 were used. Fibroblast and keratinocyte isolation from human foreskin was approved by the ethics committee of the medical faculty of the University Tübingen (093/2019B02). Keratinocyte cell lines N/TERT1 or N/TERT2G were cultured in tissue flasks in CnT-BM.1 Basal Medium 1 with supplements at 37°C and 5% CO_2_. 2-3 days before using the keratinocytes for experiments, keratinocytes were cultured in CnT-Prime Epithelial Proliferation Medium. R2F/TERT fibroblast cell line was cultured in tissue flasks in Human Fibroblast Expansion Basal Medium (Gibco) and Medium 199 (Gibco) (1:1) supplemented with iron-supplemented fetal calf serum (FCS) (Sigma-Aldrich), 10 ng/ml epidermal growth factor (Sigma-Aldrich) and 0.4 µg/ml hydrocortisone (Sigma-Aldrich). Culture conditions were at 37°C and 5% CO_2_. N/TERT1, N/TERT2G and R2F/TERT cell lines were kindly provided by J. G. Rheinwald (Harvard Medical School, Boston, USA) (Dickson et al., 2000).

### Naïve CD4^+^ T cell isolation and TH1 T cell generation

Untouched naïve human CD4^+^ T cells were isolated from PMBCs by magnetic-activated cell sorting using the Naïve CD4^+^ T Cell Isolation Kit II (Miltenyi) and an LS Column (Miltenyi) according to the manufacturer's protocol. TH1 T cell polarization was induced by using 5 µl Dynabeads Human T-Activator CD3/CD28 (Gibco) with 1×10^6^ cells, 10 ng/ml human IFN-γ (Peprotech), 10 ng/ml human IL-12 (Peprotech) and 1 µg/ml anti-IL-4 (Peprotech). T cells were cultured for 14 days in RPMI medium containing L-glutamine and 25 mM HEPES (Gibco) supplemented with 10% FCS, 1% penicillin-streptomycin (Thermo Fisher Scientific) and 0.02 mM sodium pyruvate (Thermo Fisher Scientific) at 37°C and 5% CO_2_.

### Bacterial strains and culture conditions

*Staphylococcus aureus* USA 300 LAC was used for infection experiments in this study. Bacteria were aerobically grown in tryptic soy broth medium at 37°C and orbital shaking. Infection was performed with logarithmically growing bacteria (optical density=0.5).

### Generation of 3D HSEs

#### 3D HSEs based on a fibroblast matrix

To generate the fibroblast matrix, we used the protocol described by Berning et al. (2015) except that we used CnT-Prime Fibroblast Proliferation Medium instead of Dulbecco's modified Eagle medium with 10% FCS (medium 1). In detail, 5×10^5^ fibroblasts in 500 µl CnT-Prime Fibroblast Proliferation Medium supplemented with 200 µg/ml 2-phospho-L-ascorbic acid trisodium salt (Sigma-Aldrich) were directly seeded onto 12-well inserts (0.4 μm, Greiner Bio-One) at days 1, 3 and 5. CnT-Prime Fibroblast Proliferation Medium supplemented with 200 µg/ml 2-phospho-L-ascorbic acid trisodium salt was added in the lower well, and the dermal compartment was cultured at 37°C for at least 4 weeks. Subsequently, the medium in the upper well was removed and 2.5×10^5^ keratinocytes in 500 µl CnT-Prime Epithelial Proliferation Medium supplemented with 200 µg/ml 2-phospho-L-ascorbic acid trisodium salt were seeded on top of the dermis. Concurrently, the culture medium in the lower well was changed to CnT-Prime Epithelial Proliferation Medium supplemented with 200 µg/ml 2-phospho-L-ascorbic acid trisodium salt, and the HSEs were cultured at 37°C for an additional 5-7 days. To induce stratum corneum formation, HSEs were then airlifted for 7-10 days only by adding CnT-Prime Airlift Medium (CELLnTEC) supplemented with 200 µg/ml 2-phospho-L-ascorbic acid trisodium salt to the lower well. During the total generation procedure, media were changed twice a week.

#### 3D HSE generation using collagen

Firstly, an acellular collagen layer was produced by adding 250 µl or 1 ml of 1.35 mg/ml neutralized (pH 7.2-7.4) collagen I (Corning) and CnT-Prime Fibroblast Proliferation Medium to 12-well or 6-well inserts (0.4 μm, translucent, Greiner Bio-One), respectively. After 2 h of incubation at 37°C, 2×10^5^ or 8×10^5^ fibroblasts were suspended in 500 µl or 3 ml CnT-Prime Fibroblast Proliferation Medium with 1.35 mg/ml neutralized (pH 7.2-7.4) collagen I and were seeded on top of the collagen matrix. After a further 4-5 h of incubation at 37°C, CnT-Prime Fibroblast Proliferation Medium was added in the upper and lower wells, and the dermal compartment was cultured at 37°C for 7 days. Subsequently, the medium in the upper well was completely removed and 2.5×10^5^ or 1.5×10^6^ keratinocytes in 10 µl CnT-Prime Epithelial Proliferation Medium were pipetted directly onto the dermis. After 5 h of incubation, the upper well was filled up with CnT-Prime Epithelial Proliferation Medium. Concurrently, the culture medium in the lower well was changed to CnT-Prime Epithelial Proliferation Medium and the HSEs were cultured at 37°C for an additional 5-7 days. From then on, HSEs were airlifted by only adding CnT-Prime Airlift Medium in the lower well. After 7-10 days in the airlift phase, 3D HSEs were used for experiments.

#### Psoriasis-like 3D skin models induced by TH17 cytokines

To induce a psoriasis phenotype, we optimized published protocols (Smits et al., 2017). To induce a psoriasis phenotype, HSEs based on a collagen or fibroblast matrix were treated for 3-5 days with 30 ng/ml IL-17A (Peprotech) and 30 ng/ml IL-22 (Peprotech) after 5-7 days during the airlift phase.

#### Psoriasis-like 3D skin models induced by integrated type 1 T cells

Immunocompetent psoriasis-like 3D skin models were established by integrating TH1-polarized T cells into HSEs. Therefore, 1×10^6^ TH1 cells in 500 µl CnT-Prime Epithelial Proliferation Medium supplemented with 10 ng/ml IL-2 were seeded onto skin constructs using a fibroblast matrix 1 day before the start of the airlift phase. The lower well was filled up with CnT-Prime Epithelial Proliferation Medium. Airlifting was than performed by only adding CnT-Prime Airlift Medium supplemented with 10 ng/ml IL-2 in the lower well. After 10 days in the airlift phase, HSEs were analyzed for experiments.

#### AD-like 3D skin models induced by TH2 cytokines

To induce an AD phenotype, we optimized published protocols (Smits et al., 2017). HSEs based on a collagen or fibroblast matrix were treated for 3-5 days with 20 ng/ml IL-4, 10 ng/ml IL-5 (Peprotech) and 10 ng/ml IL-13 (Peprotech) during the airlift phase.

#### Treatment of AD and psoriasis models

After a 10-day airlift period, HSEs based on a fibroblast matrix were treated with 2 µM tofacitinib (JAK inhibitor) (Selleck Chemicals) for 4 days. Thereafter, tofacitinib was added to CnT-Prime Airlift Medium in the lower well. Tofacitinib was added fresh after each medium change.

### Infection of 3D skin models with *S. aureus* and adhesion and invasion assay

HSEs based on a collagen matrix were infected with 1×10^8^ bacteria by topically applying bacteria for 3 h onto the epidermis using 8 mm filter paper discs (Smart Practice). To determine *S. aureus* colonization, adhesion and invasion assays were performed. Thereafter, HSEs were washed with PBS and then homogenized with a scalpel. Serial dilutions of the washing solution (to quantify loosely attached bacteria) and the homogenate (to quantify infiltrated bacteria) were plated onto blood agar plates. After overnight incubation at 37°C, colony-forming units were counted.

### RNA isolation and cDNA synthesis

Total RNA was extracted using the Nucleospin RNA Kit (Macherey-Nagel) according to the manufacturer's protocol. Complementary DNA was synthesized using the Reverse Transcriptase Kit (Thermo Fisher Scientific) with 1 μg of RNA, 5× RT buffer, Maxima reverse transcriptase (200 U/ml), 100 μM random hexamer primer and 10 mM dNTPs.

### qRT-PCR

Quantitative reverse transcription polymerase chain reaction (qRT-PCR) was performed using the SYBR Green PCR Master Mix (Thermo Fisher Scientific) and analysis was performed on a LightCycler 96 (Roche Life Science). Relative expression of target genes was determined using the comparative C_T_ method. The housekeeping genes 18S, actin or *GAPDH* were used. The primers used are listed in in Table S1. Unpaired two-tailed *t*-tests and visualization of statistical data were performed using GraphPad Prism (version 8.0.1 for Windows, GraphPad Software, San Diego, CA, USA; https://www.graphpad.com/).

### Immunohistochemistry

Skin reconstructs were fixed with 4% paraformaldehyde overnight, dehydrated and embedded in paraffin. For immunohistochemical staining, 3 μm-thick tissue sections were obtained using a HM325 manual microtome (Thermo Fisher Scientific) and deparaffinized. For elafin and filaggrin staining, sections were pretreated in a pressure cooker with citrate buffer at pH 6 for 9 min. For CD3 staining, sections were pretreated in a pressure cooker with EDTA buffer at pH 8 for 15 min. Afterwards, tissue sections were blocked in 5% donkey serum in PBS containing 0.05% Triton X-100 for 90 min and subsequently incubated with the respective antibody overnight at 4°C. The antibodies used are listed in in Table S2. The next day, tissue sections were first incubated with primary enhancer (Lab Vision UltraVision LP Detection System, Thermo Fisher Scientific) for 20 min at room temperature and then incubated with AP polymer (Lab Vision UltraVision LP Detection System) for 30 min at room temperature. Staining was subsequently performed using the Lab Vision Liquid Fast Red Substrate System (Thermo Fisher Scientific) according to the manufacturer's instructions. Afterwards, Hematoxylin and Eosin (H&E) staining (Agilent/Dako) was performed for 1.5 min.

### Antibody–oligonucleotide conjugation and CODEX-tagged antibody tissue staining

CODEX was performed as previously described (Schürch et al., 2020). Paraffin blocks of collagen-based skin model samples were evaluated by H&E staining and the best epidermal regions were selected for preparation of a tissue array, including five samples of untreated skin models, five samples of cytokine models for the AD phenotype and five skin models for the psoriasis phenotype. The tissue array was then cut into 4 µm-thick slices and mounted on a Vecta-bond (Vector Labs, SP-1800)-coated cover slip for CODEX analysis.

All the indicated antibodies were conjugated to a unique DNA oligonucleotide and tested and validated beforehand. Oligonucleotide conjugation was performed as previously described (Black et al., 2021). For all details on antibodies and oligonucleotides, see Table S2.

Staining was followed by washing and fixation of the tissue for imaging. Automated image acquisition was then performed using the Akoya CODEX PhenoCycler on a Keyence BZ-X810 inverted fluorescence microscope equipped with a CFI Plan Apo l 20×/0.75 objective (Nikon). For multicycle imaging of the tissue array spots, the multipoint function of the BZ-X viewer software was manually programmed to the center of each tissue array spot. The tissue array is a paraffin block that was prepared by extracting cylindrical tissue cores from different paraffin skin model blocks and re-embedding them into a single block. This ensured a faster work process as well as identical imaging conditions for every sample. All data were processed using the CODEX processor, creating hyperstacked images of all fluorescence channels following previous background subtraction. Processed images were analyzed using ImageJ/FIJI. Overlays and single-marker pictures shown in the figures were created using custom-made scripts for combination of selected channels from focused CODEX hyperstacks. Quantification of marker intensities was performed by manual selection of epidermal regions using freehand selection and the measure tool for mean marker expression provided in ImageJ/FIJI. For further quantification of marker expression between the samples, segmentation analysis was performed using QuPath (Bankhead et al., 2017). First, the brush tool was used for annotation of the epidermis, followed by detection of cells positive for nuclear staining with DRAQ5 (Cell Signaling Technology, 4084L). Subsequently measurements for mean nuclear expressions of respective markers were sorted in Excel (Version 2306). Unpaired two-tailed *t*-tests and visualization of statistical data were performed using GraphPad Prism.

### 3D reconstruction by multiphoton microscopy

HSEs were stained with DRAQ5 (Thermo Fisher Scientific, PI62251, 1.25 µM) and Phalloidin–Alexa Fluor 546 (Thermo Fisher Scientific, A22283, 1:400) for 3 days at 4°C. Imaging of collagen-based skin models was performed on a customized multiphoton microscope (TriMScope-II, Miltenyi Biotec), equipped with two parallel laser lines (Coherent Chameleon Ultra II Titanium:Sapphire and Coherent Chameleon Discovery TPC). 3D volumes of 350×350×800 µm were acquired with a 25× Nikon CFI APO LWD Objective (1.10 NA) by sequential scanning at 1090 nm (mCherry, Phalloidin–Alexa Fluor 546, SHG; 60-100 mW) and 1280 nm (DRAQ5, THG; 60-100 mW). Images were processed using ImageJ/FIJI and Bitplane Imaris 9.9. Fluorescence and higher harmonic generation signals were bandpass filtered with 585/30 (Alexa Fluor 546), 620/60 (mCherry), 710/75 (DRAQ5), 525/50 (SHG at 1090 nm excitation) and 420/50 (THG at 1280 nm excitation) and detected with GaAsP detectors (Hamamatsu).

For presentation of images, background noise was removed using the Background Subtraction plugin in FIJI and images were scaled and adjusted for brightness, contrast and gamma to enhance visualization. Unmodified images were used for intensity quantifications.

## Supplementary Material

10.1242/dmm.050541_sup1 Supplementary information
