# Supplementary material for: Histological and functional characterization of 3D human skin models mimicking the inflammatory skin diseases psoriasis and atopic dermatitis
Source: Dis Model Mech. 2024 Jan 22;17(1):dmm050541. doi: 10.1242/dmm.050541 (PMC10846593; doi:10.1242/dmm.050541)
Supplement: Supplementary information [file dmm-17-050541-s1.pdf]

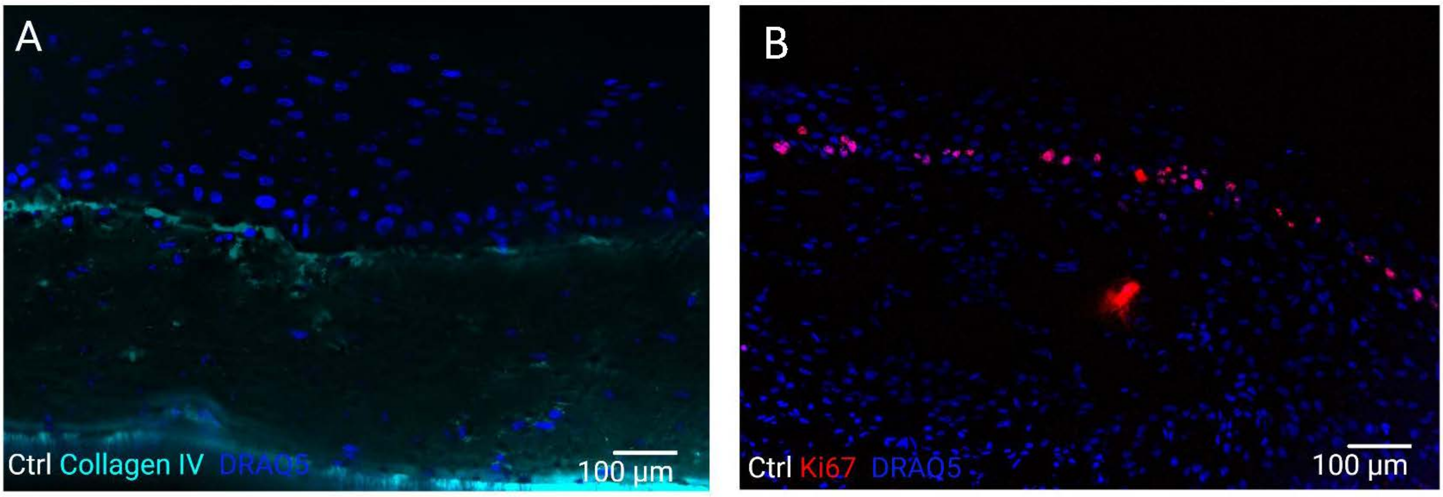

Fig. S1. Collagen IV and Ki67 staining of HSEs. (A) CODEX fluorescent image of Collagen IV expression of an HSE built up on a collagen matrix.**(B)** CODEX fluorescent image of Ki-67 expression in the same HSE. DRAQ5 CODEX fluorescent image was used as a nuclear marker. The same HSEs were used for expression analyses as for the analyses shown in Fig. 3.

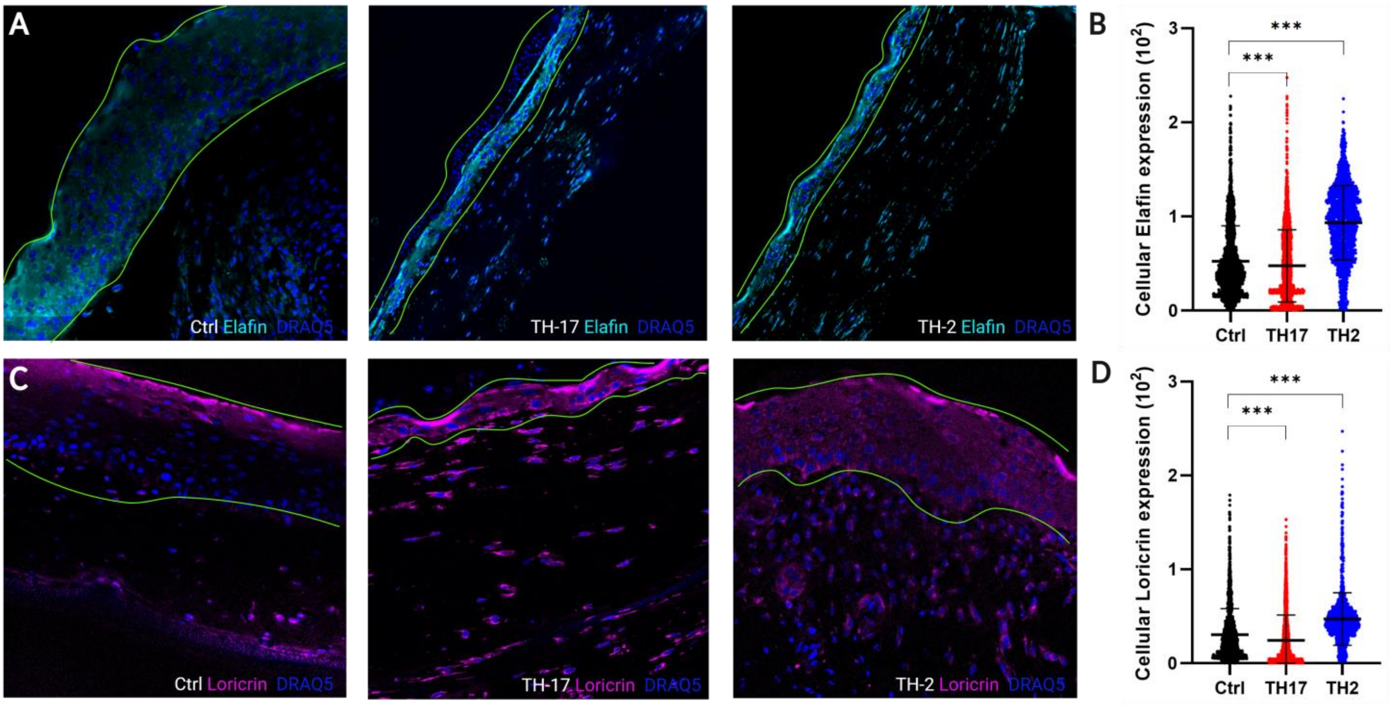

Fig. S2. Elafin and Loricrin expression in HSEs. (A) CODEX fluorescent image comparison of Elafin expression in untreated control, TH-17 and TH-2 3D human skin models. 3 day cytokine treatment, 6-well collagen matrix. Outline shows ROI for statistical analysis (epidermis green). **(B)** Statistical comparison of Elafin expression per epidermal cell by unpaired t-test. **(C)** CODEX fluorescent image comparison of Loricrin expression in untreated control, TH-17 and TH- 2 3D human skin models. 3-day cytokine treatment, 6-well collagen matrix. Outline shows ROI for statistical analysis (epidermis green). **(D)** Statistical comparison of Loricrin expression per epithelial cell by unpaired t-test. (A-D) The same HSEs were used for expression analyses as for the analyses shown in Fig. 3.

Disease Models & Mechanisms • Supplementary information

Table S1. Primer sequences used for qRT-PCR

**Target**	**Sequence (5´→3´)**
Elafin forward	catgagggccagcagctt
Elafin reverse	tttaacaggaactcccgtgaca
Filaggrin forward	acttcactgagtttcttctgatggtatt
Filaggrin reverse	tccagacttgagggtctttttctg
Loricrin forward	ccggagatggtggccttctctct
Loricrin reverse	ggcctgatgtgagttgccatgct
hBD1 forward	atggcctcaggtggtaactttc or
	ggtaactttctcacaggccttgg
hBD1 reverse	cacttggccttccctctgtaac or
	tccctctgtaacaggtgccttg
hBD2 forward	gatgcctcttccaggtgtttt or
	ataggcgatcctgttacctgcc
hBD2 reverse	ggatgacatatggctccactctt
	catcagccacagcagcttcttg
LL-37 forward	ccaggcccacgatggat
LL-37 reverse	accagcccgtccttcttga
IL-1alpha forward	ggttgagtttaagccaatcca
IL-1alpha reverse	tgctgacctaggcttgatga
IL-1beta forward	ctgtcctgcgtgttgaaaga
IL-1beta reverse	ttgggtaatttttgggatctaca
IL-6 forward	caggagcccagctatgaact
IL-6 reverse	gaaggcagcaggcaacac
CXCL-10 forward	ggtgagaagagatgtctgaatcc
CXCL-10 reverse	gtccatccttggaagcactgca
Actin-forward	caccattggcaatgagcggttc
Actin-reverse	aggtctttgcggatccacgt
GAPDH forward	gtctcctctgacttcaacagcg
GAPDH reverse	accaccctgttgctgtagccaa
18S forward	cggctaccacatccaaggaa
18S reverse	gctggaattaccgcggct

Disease Models & Mechanisms • Supplementary information

Table S2. Antibodies and oligonucleotides

Antibodies used for immunohistochemistry

Target protein	Clone	Dilution	Supplier
CD3	MRQ-39	1:200	Cell Marque (#103R-94)
Elafin	H-2	1:100	Santa Cruz (#sc-398075)
Filaggrin	AE21	1:100	Santa Cruz (#sc-80609)
Ki-67	MIB-1	1:100	Dako (#M7240)
Loricrin	Poly19051	1:500	Biolegend (#905103)

Antibodies used for CODEX

Antibody	Clone(s)	Manufacturer	Cat #	Oligo
Loricrin	Poly19051	Biolegend	905104	A71
Elafin	H-2	SantaCruz	sc-398075	A80
Filaggrin	AE21	SantaCruz	sc-80609	A26
Collagen IV	polyclonal	Abcam	Ab6586	A33
Ki67	B56	BD Biosciences	556003	A6
S. aureus	poly	Novus	NB100-64499	A11
NOS2	polyclonal	Novus	NB300-605	A74

Oligonucleotides used for CODEX

Oligo	Fluorescent bottom oligo sequences (5'-3')	Short top oligo sequences (5'-3')
A6	/5Alex647N/ATCGTAACACATCCA	/mal/TGGATGTGTTACGAT
A11	/5Alex647N/ACGAGTGTATAACCC	/mal/GGGTTATACACTCGT
A26	/5ATTO550N/TTGGTTAGACAAGTG	/mal/CACTTGTCTAACCAA
A33	/5Alex647N/CGCTCCTCATGATAA	/mal/TTATCATGAGGAGCG
A71	/5ATTO550N/GCTGGGGGTGA	/mal/TCACCCCCAGC
A74	/5ATTO550N/TCGTCGAAGCAAAT	/mal/ATTTGCTTCGACGA
A80	/5ATTO550N/TGGTGCCCCG	/mal/CGGGGCACCA

Disease Models & Mechanisms • Supplementary information
